# Clinical features and prognosis of patients co-infected with HIV and tuberculosis in the ICU

**DOI:** 10.1186/cc12930

**Published:** 2013-11-05

**Authors:** Ana Carla Pecego, Camila Ribeiro da Silva, Rodrigo T Amâncio, Denise Medeiros, Emerson C Mesquita, André M Japiassú, Fernando Bozza

**Affiliations:** 1 Instituto de Pesquisa Clínica Evandro Chagas, FIOCRUZ, Rio de Janeiro, Brazil

## Background

Despites advances in treatment, tuberculosis (TB) remains a global health threat and is the leading co-infection among Brazilian HIV-infected patients [1-4]. Mortality of TB in the presence of sepsis and shock septic can be as high as 67%, with respiratory failure being the leading cause of ICU admission [2,3,5]. Clinical factors that enhance mortality among HIV-TB patients are yet to be explored.

## Materials and methods

We retrospectively accessed data of HIV/AIDS critically ill patients from January 2007 until May 2012, at a referral infectious diseases hospital. All patients admitted to the ICU with laboratory-confirmed TB were included in the analysis. Demographic and clinical data were categorized for survivors and nonsurvivors and the results were displayed as frequency (%), median values and interquartile range.

## Results

Fifty patients were included. Hospital mortality was 48%. Age was 31.5 years (26.5 to 42.75) in the survivors group versus 33.5 years (30 to 45.25) in nonsurvivors (*P *= 0.25), and the SAPS II score was 44.5 (37.25 to 55.75) versus 47.25 (38.75 to 54.25) (*P *= 0.58). The most common TB presentation was disseminated disease (56%) followed by pulmonary (44%), with no difference according to survival. The delta of days between ICU admission and beginning of TB treatment was not different between groups. Rifampicin (94%), pyrazynamide (94%), isoniazide (92%) and ethambutol (76%) were administered in the majority of patients, while fluoroquinolones and aminoglycosides were administered in 64% and 54% respectively. Nonsurvivors presented with more elapsed time since HIV diagnosis (1 (1 to 7) vs. 26 (5 to 72), *P *= 0.10); lower nadir CD4 cell count (72.5 (27.5 to 133.5) vs. 24 (14.5 to 63), *P *= 0.03); and HAART initiated within 30 days after admission (62% vs. 29%, *P *= 0.03, odds ratio 3.9 (95% CI 1.2 to 12.7)). The main reason for ICU admission was respiratory failure (70%). Nonsurvivors needed mechanical ventilation (88% vs. 48%, *P *= 0.006) and vasopressors (71% vs. 41%, *P *= 0.05) more frequently. Neurological dysfunction was more common in nonsurvivors (79% vs. 41%, *P *= 0.01, odds ratio 5.2 (95% CI 1.5 to 18.2)). After multivariate analysis, neurological dysfunction was associated with higher mortality, while HAART in the first 30 days of hospitalization was a protective associated factor.

## Conclusions

The disseminated form was the most common presentation of TB in HIV/AIDS critically ill patients. Nonsurvivors were more prone to multiple organ dysfunction syndrome, with neurological dysfunction associated with hospital mortality. The administration of HAART within 30 days of hospitalization was associated with survival.

## References

[B1] Pacheco AG Duronvi B Cavalcante SC AIDS-related tuberculosis in Rio de Janeiro, Brazil Plos One 2008 17 3132 10.1371/journal.pone.0003132 PMC2518952 18781195

[B2] Silva DR Menegotto DM Schulz LF Mortalitity among patients with tuberculosis requiring intensive care: a retrospective cohort study BMC Infect Dis 2010 17 54 10.1186/1471-2334-10-54 20205952 PMC2843613

[B3] Zahar J-R Azoulay E Klement E Delayed treatment contributes to mortality in ICU patient with severe active pulmonary tuberculosis and acute respiratory failure Intensive Care Med 2001 17 513 520 10.1007/s001340000849 11355119 PMC7095425

[B4] Powell K Huang L The art of caring for patients with of HIV infection at the ICU Intensive Care Med 2009 17 1659 1661 10.1007/s00134-009-1578-1 19636534 PMC2749176

[B5] Japiassú AM Amâncio RT Mesquita EC Medeiros D Bernal HB Nunes EP Luz PM Grinsztejn B Bozza F Sepsis is a major determinant of outcome in critically ill HIV/AIDS patients Crit Care 2010 17 R152 10.1186/cc9221 20698966 PMC2945136

